# *Cichorium intybus* L. Hairy Roots as a Platform for Antimicrobial Activity

**DOI:** 10.3390/ph16020140

**Published:** 2023-01-18

**Authors:** Suvi T. Häkkinen, Katarina Cankar, Liisa Nohynek, Jeroen van Arkel, Markus Laurel, Kirsi-Marja Oksman-Caldentey, Bart Van Droogenbroeck

**Affiliations:** 1 VTT Technical Research Centre of Finland Ltd., Tietotie 2, 02044 Espoo, Finland; 2 Wageningen Plant Research, Wageningen University & Research, 6708 PB Wageningen, The Netherlands; 3 ILVO Institute for Agriculture, Fisheries and Food Research, 9090 Melle, Belgium

**Keywords:** chicory, antimicrobial activity, hairy roots, sesquiterpenes, polyphenols, MRSA

## Abstract

Industrial chicory is an important crop for its high dietary fibre content. Besides inulin, chicory taproots contain interesting secondary metabolite compounds, which possess bioactive properties. Hairy roots are differentiated plant cell cultures that have shown to be feasible biotechnological hosts for the production of several plant-derived molecules. In this study, hairy roots of industrial chicory cultivars were established, and their potential as a source of antimicrobial ingredients was assessed. It was shown that hot water extracts of hairy roots possessed antimicrobial activity against relevant human microbes, whereas corresponding chicory taproots did not show activity. Remarkably, a significant antimicrobial activity of hot water extracts of chicory hairy roots towards methicillin-resistant *Staphylococcus aureus* was observed, indicating a high potential of hairy roots as a host for production of antimicrobial agents.

## 1. Introduction

Chicory (*Cichorium intybus* var. *sativum* L.) is an industrial crop species cultivated for the production of inulin, which is a fructose polymer used as a low-calorie sweetener and prebiotic [1]. The phytochemicals are mainly present in the chicory root. Chicory is also well-known for its bitter taste associated with the presence of sesquiterpene lactones (STLs). The most abundant STLs, lactucin, 8-deoxylactucin, and lactucopicrin, and their oxalate and 11β,13-dihydro -derivatives are based on a guaianolide skeleton [2]. Of these, lactucopicrin and dihydrolactucopicrin are more bitter than quinine hydrochloride [3] and they have a role as phytoalexins in plant defence [4]. Chicory also produces typical polyphenols (PPs), which have biological relevance [5]. Recently, different types of chicory extracts were comprehensively assessed for their bioactive potential [6]. Besides possessing antibacterial and antifungal activities towards a range of human pathogenic microbes, remarkably, the chicory supercritical fluid extract and a purified fraction thereof were able to inhibit the growth of antibiotic-resistant strains of *Staphylococcus aureus* (MRSA) and *Pseudomonas aeruginosa* (AMP_R_), which are both currently considered as major global health threats. In addition, compared with pure sesquiterpene lactones, chicory extracts showed higher antibiofilm activity against the yeast *Candida albicans* [6].

Hairy roots are a valuable biotechnological tool for studying the molecular mechanisms of a number of basic phenomena in plant behavior, biochemistry, and physiology. The main targets of hairy root culture-related applications include biotransformation, production of high-value plant metabolites, and phytoremediation [7,8,9]. Being differentiated cells, hairy roots have often shown to possess higher production capacities for plant secondary compounds compared with undifferentiated cells. This makes them interesting tools for both research purposes as well as for actual production hosts [10]. Various high-value plant secondary metabolites have been produced by differentiated plant tissues successfully in bioreactors up to 10 m^3^ in scale [11,12].

In this study, the potential of using *C. intybus* hairy roots for the production of antimicrobial extracts was assessed. Two different root chicory cultivars were used to induce hairy root cultures. The growth and bioactivity were followed during the cultivation, and typical chicory-derived STLs and PPs were analysed. Antimicrobial activity towards typical human skin microbes was assessed in different growth stages.

## 2. Results

### 2.1. VL-70 Hairy Roots

Altogether 13 hairy root clones deriving from the VL-70 plant were established. Biomass was measured after 28 d of cultivation. Hairy roots accumulated biomass at 13.2–17.3 g DW/L except for one clone, which displayed significantly lower biomass compared with the other clones, i.e., 3.8 g DW/L. Seven of these VL-70 hairy root clones were analysed for their STL and PP production (Table 1). The most abundant STL was lactucopicrin 15-oxalate, followed by other oxalates and 8-deoxylactucin. The lowest amounts were found for lactucin and lactucopicrin. There were no clear indications of a particular clone having high overall STL accumulation; however, the production of STLs in V_10 in general was low, while PPs accumulated in higher amounts compared with the other clones. Isochlorogenic acid represented the highest abundant PP, followed by chlorogenic acid and chicoric acid. The highest production of PPs were observed with clones V_10 and V_15.

### 2.2. K1793 Hairy Roots

Altogether, five well-growing hairy root lines were established from the K1793 plant. The biomass varied from 11.8 g to 15.1 g DW per liter, showing on average 14% lower biomass accumulation than VL-70 hairy roots, at day 28. K1793 hairy roots showed lower chlorogenic acid, isochlorogenic acid, and lactucopicrin accumulation; furthermore, levels were 30%, 30%, and 20% compared with those of VL-70 clones, respectively (Table 2). Within K1793 hairy roots, clone K_7 was a particularly high producer of 8-deoxylactucin and lactucin 15-oxalate. VL-70 lines in general accumulated somewhat higher levels of the studied compounds (Table 1 and Table 2).

In general, between the two taproots, K1793 taproots accumulated higher amounts of STLs and PPs, except for chicoric and chlorogenic acids (Table 3). A remarkably high amount of oxalates were accumulating in K1793 taproots, being more than 10-fold in the case of 15-oxalates of lactucopicrin and 8-deoxylactucin, and almost 200-fold in the case of lactucin oxalate. The levels of STLs and PPs are in range with our recent study, where taproots of field-cultivated Belgian endives were analysed [5] However, isochlorogenic acid A contents in the current study with industrial chicory taproots were clearly higher than in Belgian endive.

### 2.3. Bioactivity Assessment

**Figure 1:**
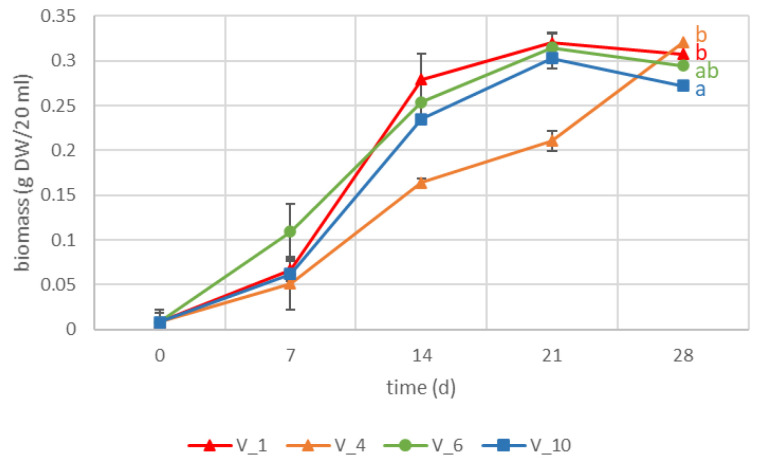
Growth curves of four VL-70 hairy root clones. Data represents mean ± stdev of three biological replicates. Letters indicate statistical differences (*p* < 0.05).

Four hairy root clones showing distinctive secondary metabolite accumulation patterns V_1, V_4, V_6, and V_10 were cultivated in liquid cultures in triplicate, and biomass accumulation was followed during 28 d (Figure 1). Clones V_1, V_6, and V_10 had similar growth patterns, whereas V_4 showed slower growth, however reaching a high biomass at day 28. Clone V_10 differed statistically from high accumulating clones V_1 and V_4 in its biomass accumulation at the end of cultivation period, remaining at 15% lower than V_1.

**Figure 2:**
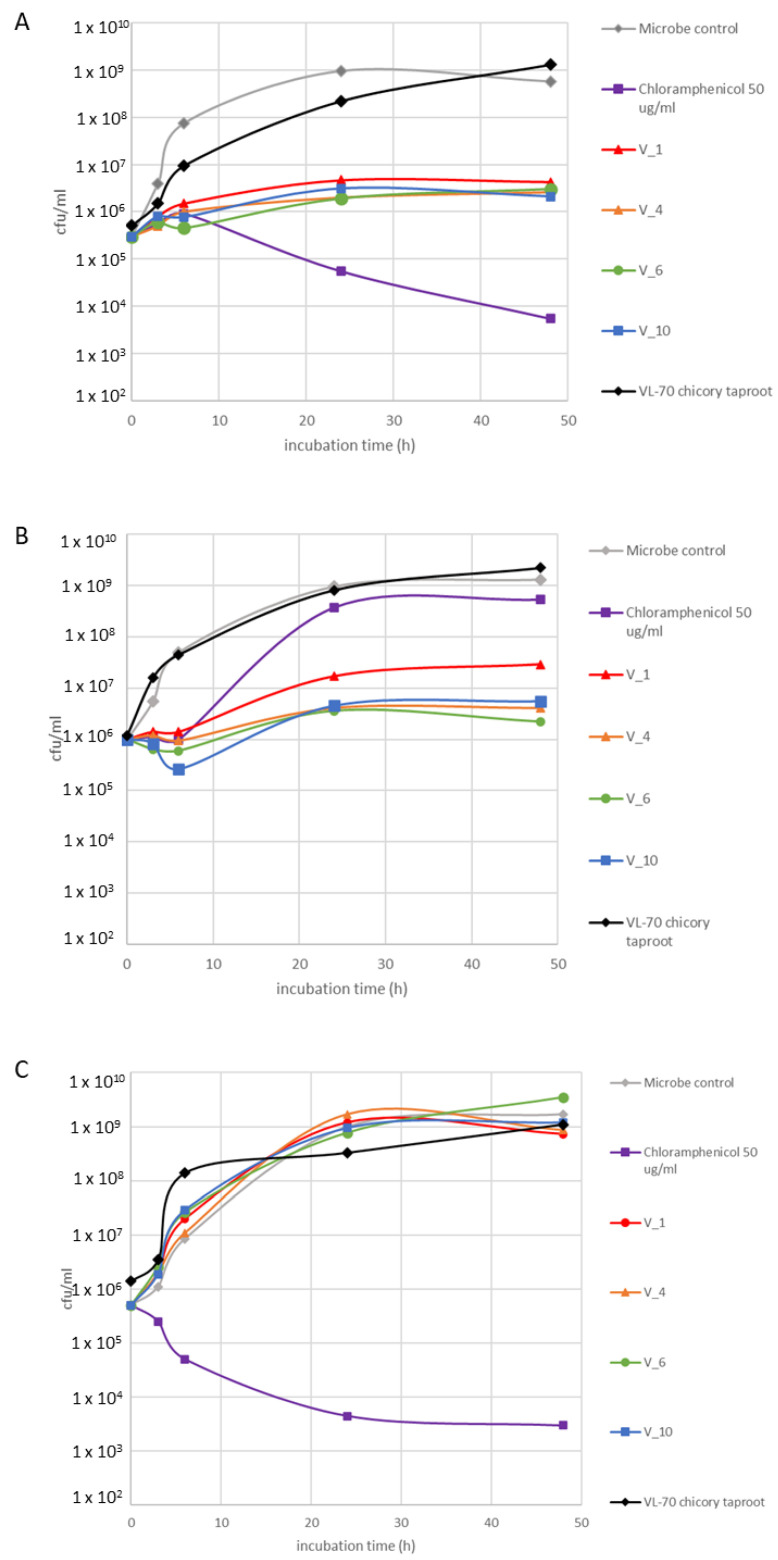
Antimicrobial activity of four VL-70 hairy root (28 d) hot water extracts (5 mg/mL) against (**A**) *S. aureus* VTT E-70045, (**B**) *S. aureus* MRSA VTT E-183582, and (**C**) *P. aeruginosa* VTT E-84219.

Antimicrobial activity of the four hairy root clones were assessed after 28 days of cultivation together with VL-70 taproot, against *S. aureus*, *S. aureus* MRSA, and *P. aeruginosa* (Figure 2). It was observed that all clones possessed moderate antimicrobial activity against *S. aureus* (Figure 2A) and also against resistant-strain *S. aureus* MRSA, with especially high activity with V_4, V_6, and V_10 (Figure 2B and Figure S1). None of the clones showed activity towards Gram-negative *P. aeruginosa* (Figure 2C). VL-70 taproot did not show antimicrobial activity against any tested bacteria.

**Figure 3:**
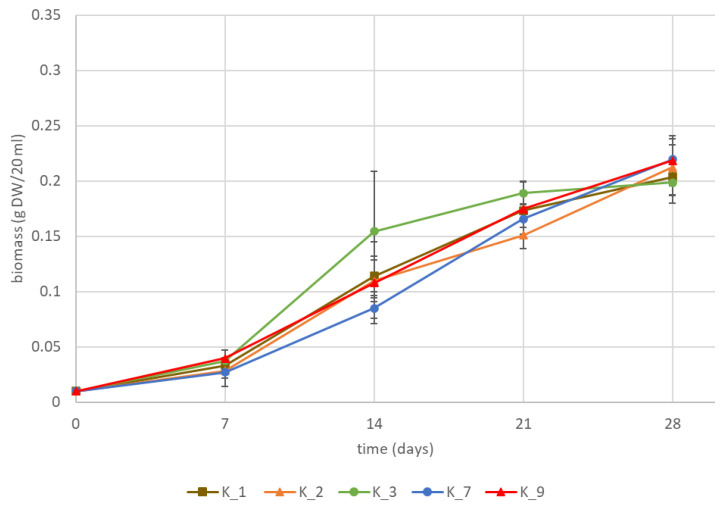
Growth of five K1793-derived hairy root clones during the 28 d cultivation period. Data represents mean ± stdev of three biological replicates.

**Figure 4:**
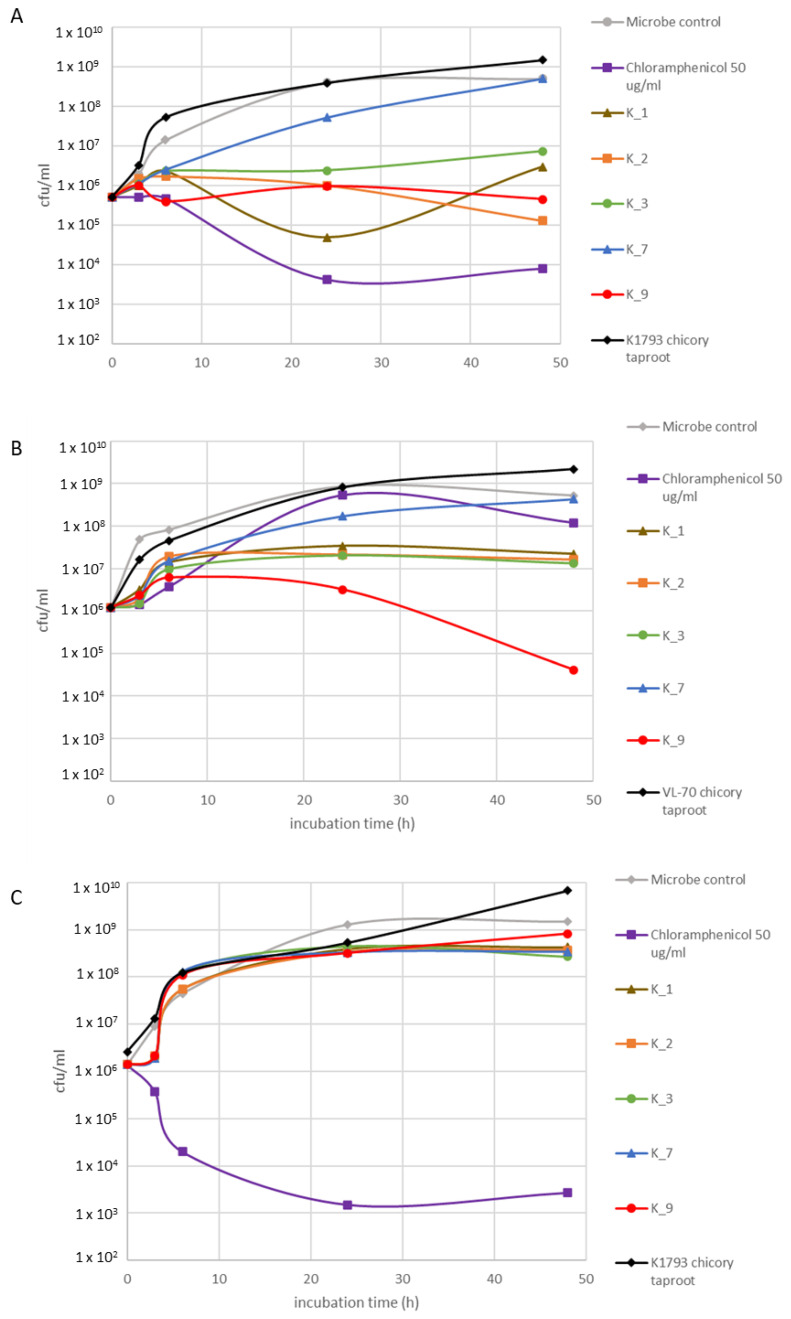
Antimicrobial activity of five K1793 hairy root (28 d) hot water extracts (5 mg/mL) against (**A**) *S. aureus* VTT E-70045, (**B**) *S. aureus* MRSA VTT E-183582, and (**C**) *P. aeruginosa* VTT E-84219. Experiment was performed twice.

Growth curves of K1793 hairy root clones are presented in Figure 3. Compared to VL-70 clones, K1793 exhibited lower biomass accumulation with on average 0.21 g DW compared to VL-70 average 0.30 g DW. There were no major differences between the growth patterns of individual clones and the biomass reached at day 28 (*p* < 0.05). Antimicrobial activity was assessed against the same bacteria as for VL-70 clones (Figure 4). In addition, the activity of K1793 chicory taproot was examined. As with VL-70 hairy roots, K1793 hairy roots also exhibited moderate to strong antimicrobial activity, except K_7, against *S. aureus* (Figure 4A). Particularly high activity was observed with clones K_2 and K_9. As with VL-70 hairy roots, K1793 hairy roots were also active against resistant *S. aureus* MRSA, with a very strong activity with K_9 (Figure 4B). As with VL-70 hairy roots, K1793 lines did not inhibit the growth of *P. aeruginosa* (Figure 4C). K1793 taproot did not have activity against any of the bacteria assessed.

**Figure 5:**
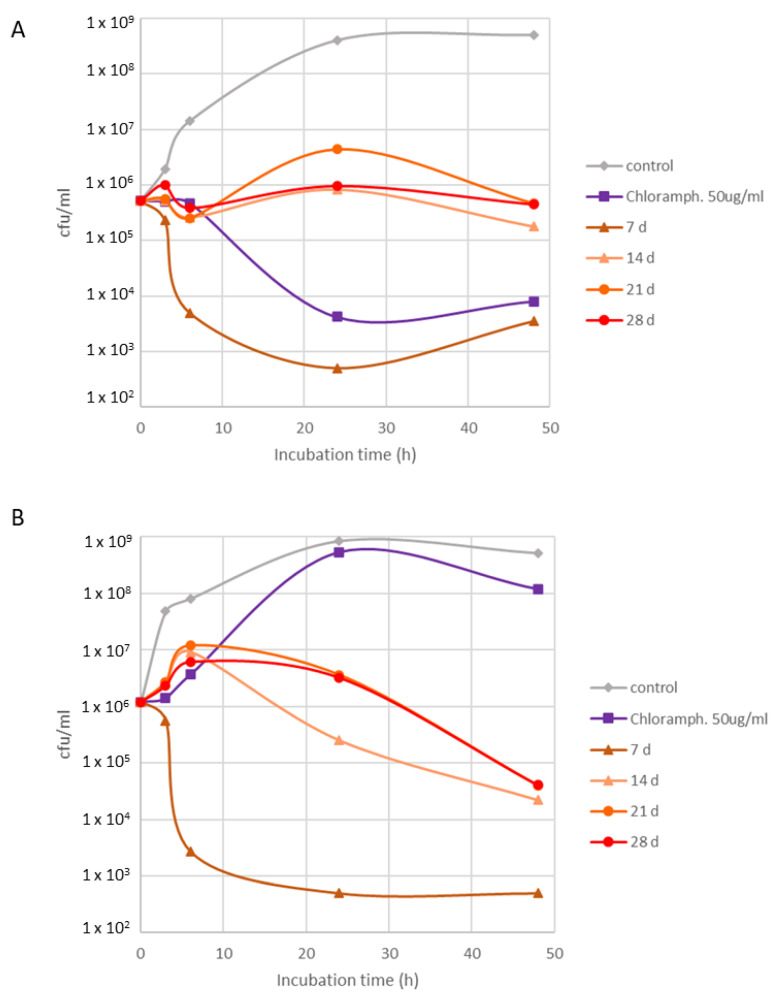
Antimicrobial activity K_9 clone assessed after 7, 14, 21, and 28 d of cultivation against (**A**) *S. aureus* VTT E-70045 and (**B**) *S. aureus* MRSA VTT E-183582. Experiment was performed twice.

The clone K_9, which showed particularly high activity, was also examined during its 28 day growth period (Figure 5). Remarkably, an early growth stage sample (7 d) displayed even stronger activity than commercial antibiotics towards *S. aureus* (Figure 5A). No differences were observed between time points 14, 21, and 28 d. Interestingly, the same 7 d sample also showed exceptionally high activity towards resistant *S. aureus* MRSA (Figure 5B). No differences in respective samples were observed against *P. aeruginosa*.

## 3. Discussion

Chicory is a highly interesting plant, being an important source for dietary fibre inulin. Besides inulin, chicory is known for its medicinal value and its metabolites have been studied for their bioactive properties, being largely attributed to typical chicory STLs. Properties range from antimicrobial, anti-inflammatory, and anti-cancer potential [6,13,14]. During industrial processing of chicory for inulin production, the terpene and polyphenol rich fraction is discarded. Recently, we showed that this industrial by-product can be used as cosmetic ingredient in an economically viable process scenario [15]. As a biotechnological production platform, hairy roots offer an interesting alternative for various plant-derived compounds, and in this study the potential of chicory hairy roots in regard to secondary metabolite-rich antimicrobial extract source was assessed. Compared with taproots, which are the commonly studied source of chicory metabolites, hairy roots showed higher antimicrobial potential. Furthermore, the antimicrobial activity of hairy roots was shown also for methicillin-resistant *S. aureus* strains. In our earlier study, we observed that water extracts of chicory taproot were less potent in their bioactivity potential compared with other extraction solvents, and especially compared with supercritical fluid extraction [6]. In the current study, we showed that hot water extracts of chicory hairy roots had antimicrobial activity while the respective taproot material did not display any activity towards the tested microbes. Hot water extraction has been shown to be a very potent method to enrich natural antimicrobials [16]. Extracts made by the hot water extraction process are solvent-free and thus suitable ingredients for various cosmetics or personal care purposes. In addition, when considering the large-scale extraction process, water-based extraction is beneficial when considering techno-economic and environmental aspects.

Between the two cultivars, VL-70-derived hairy root clones showed in general moderately higher amounts of STLs and PPs; however, the clonal differences were high. Chlorogenic acid concentration in hairy roots were remarkably higher than in taproots, being almost 10 and 4-fold in the case of VL-70 and K1793, respectively. Chlorogenic acid has been shown to possess antimicrobial activity, particularly against *S. aureus* [17,18]. It is interesting to note that a positive correlation between the content of phenolic compounds, including chlorogenic acid, and antioxidative properties in chicory taproots was recently observed [19]. Accordingly, in this study, we observed a correlation between chlorogenic acid content (in hairy roots and taproots) and antimicrobial activity against *S. aureus* and *S. aureus* MRSA, with correlation coefficients ranging from 0.72 to 0.95. Furthermore, in the current study, a remarkably high antimicrobial effect was observed with the K1793 hairy root in the early growth phase (day 7) in repeated assays (Figure 5). However, the concentration of chlorogenic acid was only measured throughout the study from stationary growth phase cultures (day 28). Thus, while chlorogenic acid is likely the responsible factor of the antimicrobial effect of the hairy roots in the current study, it is also possible that some other secondary compounds are contributing to the remarkably high antimicrobial activity in the early growth stage. On the other hand, considering the hairy root production platform and feasibility, collecting material at a very early growth stage poses a compromise between high bioactivity and low biomass yield and thus the total economic benefits would need to be considered before selecting this growth stage for production purposes.

## 4. Materials and Methods

### 4.1. Hairy Root Induction and Maintenance and Chicory Taproots

Two cultivars of industrial chicory were assessed is this study. VL-70 seeds and sterile in vitro plants of K1793 were obtained from ILVO Flanders Institute for Agriculture, Fisheries and Food Research, Melle, Belgium. Seeds of VL-70 lines were surface sterilized with 70% ethanol (2 min), after which the seeds were transferred to 5% sodium hypochlorite solution (15 min). After this, the seeds were rinsed three times with UHP H_2_O and then sown on solid Murashige and Skoog medium [20]. Hairy roots of *C. intybus* VL-70 line were initiated by *Agrobacterium rhizogenes* infection using altogether three bacterial strains: LBA9402, A4, and 15834. The agropine-type strains were A4 (kindly provided by Dr. David A. Tepfer, Versailles, France), LBA9402, and 15834 (kindly provided by Prof. Ulf Nyman, Copenhagen, Denmark). The strains LBA9402 and 15834 were cultivated in YMB (yeast mannitol broth) culture medium added with 100 ppm rifampicin. The strain A4 was cultivated in APM culture medium [21] added with 0.1 ppm biotin. Bacteria were cultivated on solid medium at +28 °C, 48 h prior to infection. Infections were performed by sterile syringe needle dipped in the bacterial mass and infecting a sterile explant.

For establishment of K1793 hairy roots, sterile in vitro plant leaves were infected with *A. rhizogenes* LBA9402 as described above. Explants were then placed on modified Gamborg B5-media [22,23] and were incubated in the dark for a 48-h co-cultivation period.

After the co-cultivation, all the explants were transferred onto bacteriological media plates with cefotaxime sodium 500mg/L to kill the excess bacteria. After 10–14 days, hairy roots started to appear on the wound site, and they were excised from the explant and cultivated on solid modified B5 medium. The presence of *rolB* and the absence of *virD* genes were confirmed by PCR as earlier described [12]. For biomass determination, 100 mg FW of hairy roots from both cultivars were weighed in 100 mL Erlenmeyer flasks and 20 mL of modified B5 medium was added. Roots were cultivated in dark as described in [23]. Hairy roots were harvested by filtering after 7, 14, 21, or 28 days of cultivation and freeze-dried before analyses. Three in vitro plants of lines VL-70 and K1793 were transferred to soil and cultivated for a period of 3 months to form a taproot. Taproots were collected and freeze-dried before analysis.

### 4.2. Metabolite Analysis

Freeze-dried tissues were grinded with mortar and pestle to fine powder. Semi polar metabolites were analysed by LC-MS [24]. To this end, 50 mg (±3 mg) of plant material was extracted using 950 µL 70% methanol supplemented with 0.13% formic acid followed by an incubation in the ultrasonic bath for 15 min. The plant debris was then separated from the extract by centrifugation at 14000 rpm in a tabletop centrifuge. LC-MS analysis was performed using the LC-PDA-LTQ-Orbitrap FTMS instrument (Thermo Scientific, Waltham, USA) equipped with an Acquity UPLC. Chromatographic separation and detection of metabolites was performed as described previously [5].

Targeted quantification of major chicory phenolic and terpene compounds was performed by comparison to a standard curve prepared from authentic standards of caftaric acid (Sigma-Aldrich Merck KGaA, Darmstadt, Germany), chicoric acid (Sigma-Aldrich, Merck KGaA, Darmstadt, Germany), isochlorogenic acid A (Sigma-Aldrich, Merck KGaA, Darmstadt, Germany), lactucin (Extrasynthese, Genay, France), and lactucopicrin (Extrasynthese, Genay, France). In case authentic standards were not available, compounds were relatively quantified and relative peak areas were presented. Metabolite analysis data were subjected to the analysis of variance (ANOVA) for statistical analysis using SPSS software (software version 25 for Windows, IBM). Tukey’s post hoc test (*p* > 0.05) was used to analyse differences between hairy root lines.

### 4.3. Antimicrobial Assessment

Freeze-dried hairy root samples (1 g DW) were grinded with ball mill (Retsch, 29 Hz, 60 s) and extracted with water (20 mL) by incubating in a water bath (80 °C, 1 h). The slurry was centrifuged (10 min, 2600 rcf) and filtered with a triple Miracloth filter. The extraction was performed twice and the collected supernatants were combined. After measuring the pH, the hot water extracts were frozen, freeze-dried, and stored in the freezer until analyses. Antimicrobial activities of the HT-extracts of hairy roots were evaluated against *Staphylococcus aureus* VTT E-70045 (ATCC 6538), *Pseudomonas aeruginosa* VTT E-84219 (ATCC 15692), and *S. aureus* MRSA VTT E-183582 (DSM 11822) using the method described in [25], with the modification of using reduced culture volumes of 1.0 mL [26]. Briefly, 5 mg of the HT-extract was weighted in a sterile Eppendorf tube of 2 mL and suspended with a bacterial inoculum of 1 mL in bacterial culture medium (Nutrient Broth, Oxoid). Bacterial cultures without extract were included as positive controls, and antibiotic chloramphenicol (50 µg/mL) as a negative control. The cultures with extracts in Eppendorf tubes were incubated at +37 °C, shaking at 150 rpm for 48 h. Samples were taken during cultivation on occasions of 0, 3, 6, 24, and 48 h for antimicrobial activity evaluation. Plate counts (cfu/mL) of the samples were measured, and the antimicrobial activity of the extract was evaluated by comparison of control growth curves with the ones with hairy root extracts.

## 5. Conclusions

The results of the present study, consisting of characterization of growth, phytochemical composition as well as antimicrobial properties of *C. intybus* hairy roots, demonstrated the potential of this biotechnological approach to obtain antimicrobial ingredients. The results of the current study indicated that hot water extraction is an efficient way to produce antimicrobial ingredients from chicory hairy roots against relevant human skin pathogens, while corresponding taproots did not show activity towards tested micro-organisms. It is interesting to note, that a significant activity against the growth of methicillin resistant *S. aureus* was observed with these extracts, highlighting their potential as interesting source of valuable antimicrobial agents and thus contributing to the global health threat of antibiotic resistance in general.

## Figures and Tables

**Table 1:** Comparison of sesquiterpene lactones and polyphenols produced in different hairy root lines derived from VL-70 after 28 days of cultivation. Metabolite quantities are expressed in mg/g dry weight or as relative abundance (AR). ANOVA and Tukey’s post-hoc test were performed to test the difference between the hairy root lines. The letters indicate significantly different groups at *p* < 0.05.

Hairy Root Line	Chlorogenic Acid (mg/g DW)	Isochlorogenic Acid A (mg/g DW)	Chicoric Acid (mg/g DW)	Lactucin (mg/g DW)	8-Deoxylactucin (AR)	Lactucopicrin (mg/g DW)	Lactucin 15-Oxalate (AR)	8-Deoxylactucin 15-Oxalate (AR)	Lactucopicrin 15-Oxalate (AR)
V_1	5.95 ± 0.71 ^ab^	17.61 ± 2.12 ^ab^	0.38 ± 0.03 ^a^	0.04 ± 0.02 ^ab^	12.41 ± 2.73 ^ab^	0.090 ± 0.045 ^a^	8.72 ± 1.75 ^bc^	17.28 ± 5.00 ^cd^	43.30 ± 8.89 ^c^
V_3	7.58 ± 1.73 ^ab^	20.11 ± 4.42 ^ab^	0.80 ± 0.20 ^ab^	0.02 ± 0.01 ^ab^	19.53 ± 2.24 ^bc^	0.089 ± 0.028 ^a^	3.75 ± 0.35 ^a^	11.48 ± 1.30 ^bc^	32.26 ± 5.73 ^bc^
V_4	5.18 ± 1.10 ^a^	14.24 ± 1.70 ^a^	0.38 ± 0.05 ^a^	0.04 ± 0.00 ^b^	11.18 ± 0.62 ^ab^	0.060 ± 0.010 ^a^	11.47 ± 1.08 ^c^	20.18 ± 0.89 ^d^	34.73 ± 0.81 ^bc^
V_6	9.25 ± 1.83 ^abc^	21.09 ± 4.01 ^ab^	0.83 ± 0.23 ^ab^	0.02 ± 0.01 ^ab^	22.14 ± 5.27 ^c^	0.084 ± 0.030 ^a^	1.77 ± 0.78 ^a^	3.81 ± 1.27 ^ab^	20.86 ± 5.98 ^ab^
V_8	6.69 ± 1.48 ^ab^	19.86 ± 1.86 ^ab^	0.39 ± 0.07 ^a^	0.03 ± 0.01 ^ab^	10.04 ± 1.01 ^a^	0.073 ± 0.014 ^a^	8.93 ± 2.38 ^bc^	20.32 ± 3.86 ^d^	43.84 ± 4.41 ^c^
V_10	13.61 ± 1.95 ^c^	28.77 ± 5.54 ^b^	1.04 ± 0.14 ^b^	0.01 ± 0.00 ^a^	9.78 ± 0.49 ^a^	0.029 ± 0.009 ^a^	2.75 ± 0.69 ^a^	2.05 ± 0.29 ^a^	14.11 ± 1.23 ^a^
V_15	10.61 ± 0.38 ^bc^	25.96 ± 0.86 ^b^	0.55 ± 0.06 ^a^	0.02 ± 0.00 ^ab^	14.60 ± 0.75 ^abc^	0.063 ± 0.012 ^a^	5.86 ± 0.13 ^ab^	11.75 ± 0.51 ^bcd^	43.18 ± 3.22 ^c^

**Table 2:** Comparison of sesquiterpene lactones and polyphenols produced in different hairy root lines derived from K1793 after 28 days of cultivation. Metabolite quantities are expressed in mg/g dry weight or as relative abundance (AR). ANOVA and Tukey’s post-hoc test were performed to test the difference between the hairy root lines. The letters indicate significantly different groups at *p* < 0.05.

Hairy Root Line	Chlorogenic Acid (mg/g DW)	Isochlorogenic Acid A (mg/g DW)	Chicoric Acid (mg/g DW)	Lactucin (mg/g DW)	8-Deoxylactucin (AR)	Lactucopicrin (mg/g DW)	Lactucin 15-Oxalate (AR)	8-Deoxylactucin 15-Oxalate (AR)	Lactucopicrin 15-Oxalate (AR)
K_1	2.31 ± 0.08 ^b^	6.04 ± 0.05 ^b^	0.71 ± 0.04 ^b^	0.01 ± 0.00 ^a^	2.69 ± 0.74 ^a^	0.008 ± 0.001 ^a^	7.71 ± 1.00 ^a^	4.62 ± 0.51 ^a^	17.91 ± 1.64 ^a^
K_2	2.09 ± 0.24 ^ab^	5.88 ± 0.23 ^b^	0.51 ± 0.11 ^a^	0.01 ± 0.00 ^a^	1.53 ± 0.12 ^a^	0.011 ± 0.002 ^a^	5.97 ± 0.36 ^a^	3.49 ± 0.15 ^a^	16.38 ± 0.70 ^a^
K_3	2.29 ± 0.12 ^ab^	6.24 ± 0.14 ^ab^	0.66 ± 0.04 ^ab^	0.01 ± 0.00 ^a^	1.92 ± 0.15 ^a^	0.012 ± 0.000 ^ab^	8.75 ± 1.93 ^a^	3.88 ± 0.07 ^a^	18.23 ± 0.72 ^ab^
K_7	2.27 ± 0.05 ^ab^	5.89 ± 0.08 ^ab^	0.97 ± 0.04 ^c^	0.02 ± 0.00 ^c^	6.22 ± 0.95 ^b^	0.016 ± 0.001 ^bc^	27.11 ± 1.22 ^b^	7.68 ± 0.74 ^b^	22.85 ± 0.25 ^b^
K_9	1.87 ± 0.08 ^a^	5.50 ± 0.05 ^a^	0.51 ± 0.04 ^a^	0.01 ± 0.00 ^b^	2.29 ± 0.38 ^a^	0.018 ± 0.002 ^c^	7.74 ± 0.36 ^a^	4.40 ± 0.50 ^a^	16.70 ± 2.54 ^a^

**Table 3:** Comparison of sesquiterpene lactones and polyphenols produced in VL-70 and K1793 taproots (N = 3). Metabolite quantities are expressed in mg/g dry weight or as relative abundance (AR). ANOVA was performed to test the difference between the two plant lines. The letters indicate significantly different groups at *p* < 0.05.

Plant Line	Chlorogenic Acid (mg/g DW)	Isochlorogenic Acid A (mg/g DW)	Chicoric Acid (mg/g DW)	Lactucin (mg/g DW)	8-Deoxylactucin (AR)	Lactucopicrin (mg/g DW)	Lactucin 15-Oxalate (AR)	8-Deoxylactucin 15-Oxalate (AR)	Lactucopicrin 15-Oxalate (AR)
VL-70	0.89 ± 0.09 ^a^	1.05 ± 0.23 ^a^	0.45 ± 0.08 ^b^	0.02 ± 0.00 ^a^	11.57 ± 4.46 ^a^	0.013 ± 0.003 ^a^	0.75 ± 0.25 ^a^	7.17 ± 0.93 ^a^	18.8 ± 7.61 ^a^
K1793	0.58 ± 0.04 ^a^	17.24 ± 4.63 ^b^	0.05 ± 0.00 ^a^	0.20 ± 0.00 ^b^	38.49 ± 4.65 ^b^	0.199 ± 0.015 ^b^	157.48 ± 10.38 ^b^	83.65 ± 10 ^b^	197 ± 5.92 ^b^

## Data Availability

Not applicable.

## Supplementary Materials

The following are available online at https://www.mdpi.com/article/10.3390/ph16020140/s1, Figure S1: Antimicrobial activity V_10 clone assessed after 7, 14, 21, and 28 d of cultivation.
