# Supplementary material for: Cichorium intybus L. Hairy Roots as a Platform for Antimicrobial Activity
Source: Pharmaceuticals (Basel). 2023 Jan 18;16(2):140. doi: 10.3390/ph16020140 (PMC9967317; doi:10.3390/ph16020140)
Supplement: Supplementary file 1 [file pharmaceuticals-16-00140-s001.zip › pharmaceuticals-2127471-supplementary.pdf]

A

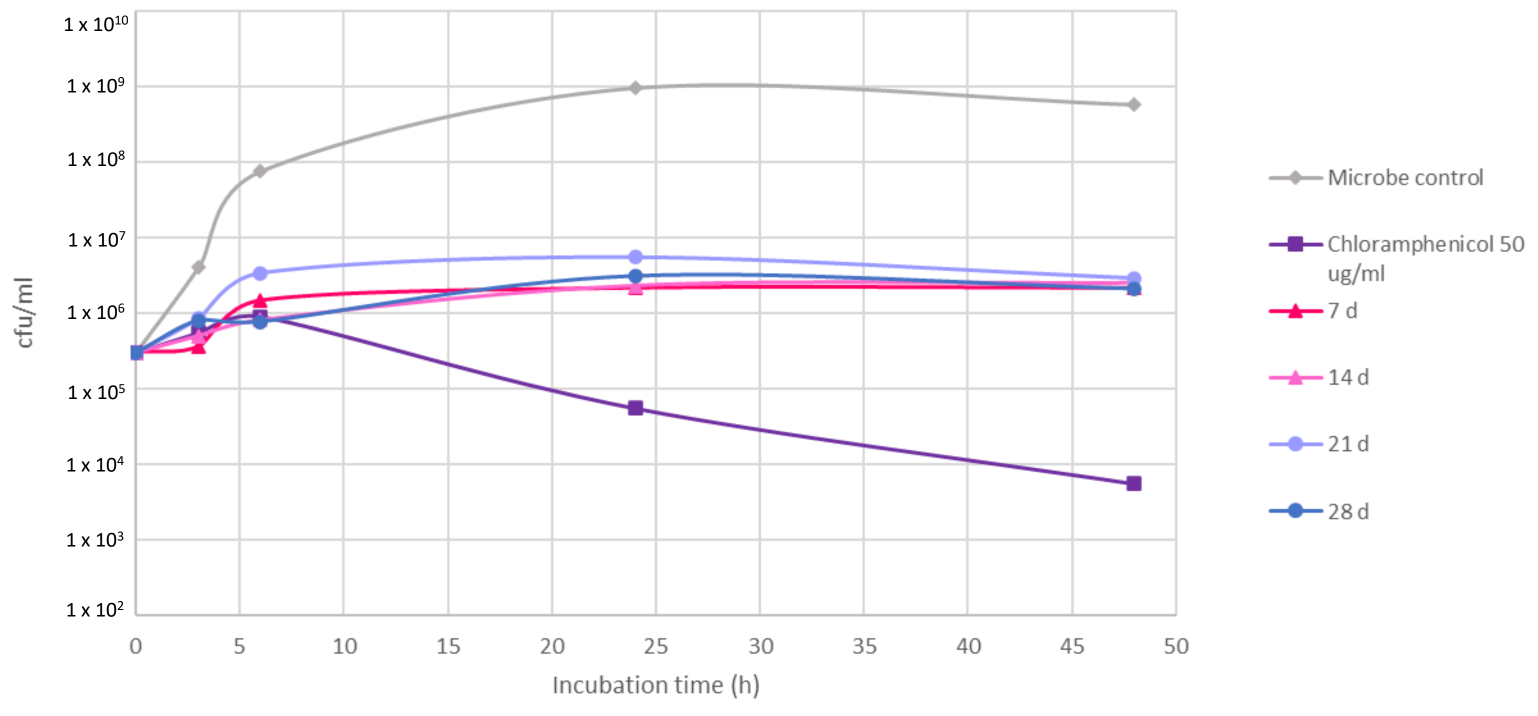

B

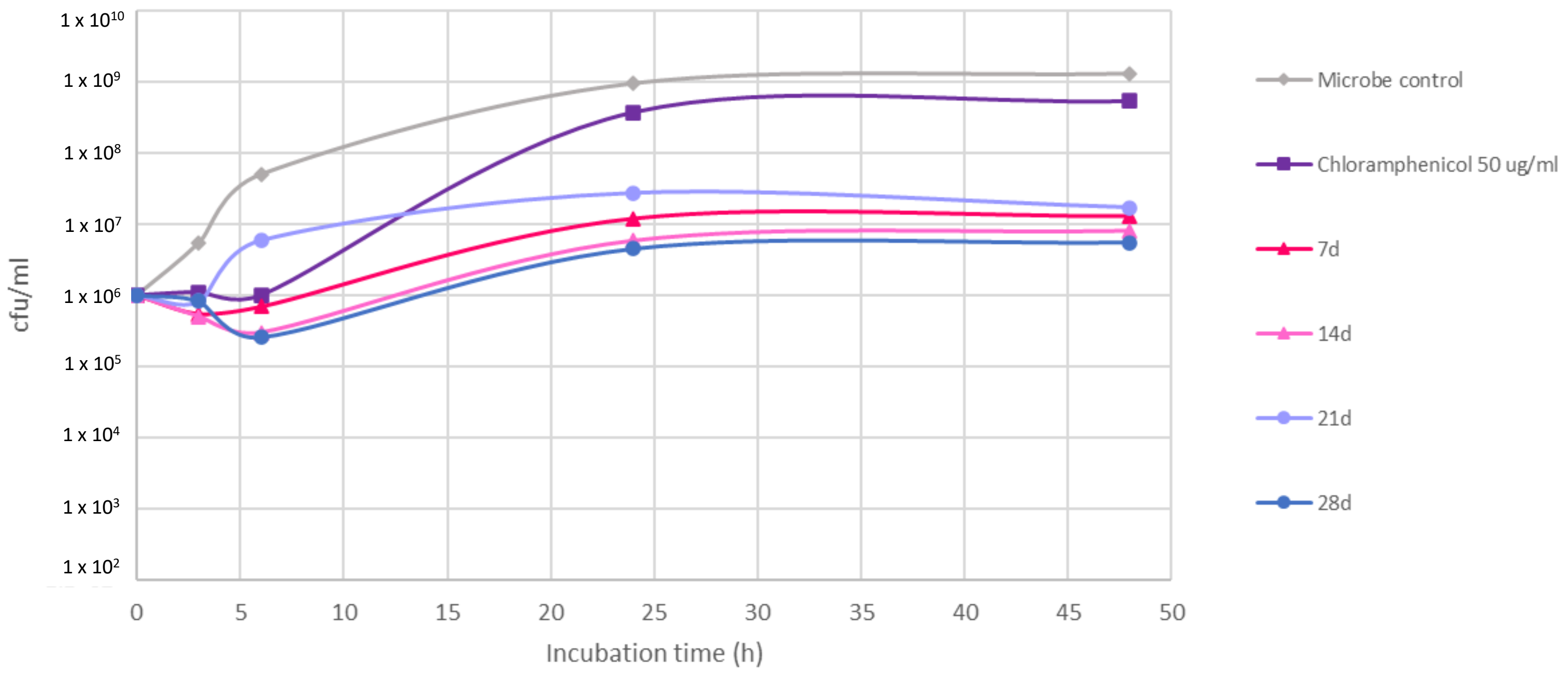

C

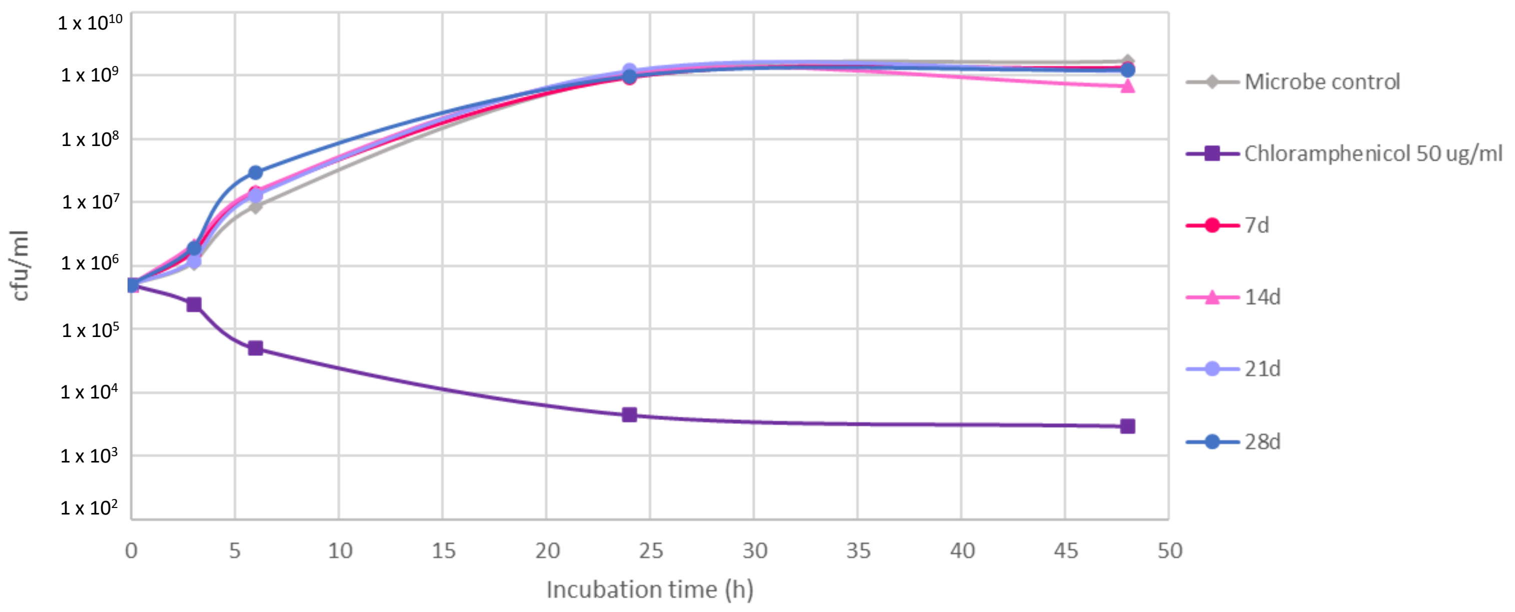

Supplementary Fig. S1. Antimicrobial activity V_10 clone assessed after 7, 14, 21 and 28 d cultivation against **A**) *S. aureus* VTT E-70045, **B**) *S. aureus* MRSA VTT E-183582 and **C**) *P. aeruginosa* VTT E-84219.
